# Differential Effects of HIF-1 Inhibition by YC-1 on the Overall Outcome and Blood-Brain Barrier Damage in a Rat Model of Ischemic Stroke

**DOI:** 10.1371/journal.pone.0027798

**Published:** 2011-11-16

**Authors:** Jingqi Yan, Bo Zhou, Saeid Taheri, Honglian Shi

**Affiliations:** 1 Department of Pharmacology and Toxicology, University of Kansas, Lawrence, Kansas, United States of America; 2 Department of Neurology, University of South Carolina, Columbia, South Carolina, United States of America; Julius-Maximilians-Universität Würzburg, Germany

## Abstract

Hypoxia-inducible factor 1 (HIF-1) is a master regulator of cellular adaptation to hypoxia and has been suggested as a potent therapeutic target in cerebral ischemia. Here we show in an ischemic stroke model of rats that inhibiting HIF-1 and its downstream genes by 3-(5'-hydroxymethyl-2'-furyl)-1-benzylindazole (YC-1) significantly increases mortality and enlarges infarct volume evaluated by MRI and histological staining. Interestingly, the HIF-1 inhibition remarkably ameliorates ischemia-induced blood-brain barrier (BBB) disruption determined by Evans blue leakage although it does not affect brain edema. The result demonstrates that HIF-1 inhibition has differential effects on ischemic outcomes and BBB permeability. It indicates that HIF-1 may have different functions in different brain cells. Further analyses show that ischemia upregulates HIF-1 and its downstream genes erythropoietin (EPO), vascular endothelial growth factor (VEGF), and glucose transporter (Glut) in neurons and brain endothelial cells and that YC-1 inhibits their expression. We postulate that HIF-1-induced VEGF increases BBB permeability while certain other proteins coded by HIF-1's downstream genes such as *epo* and *glut* provide neuroprotection in an ischemic brain. The results indicate that YC-1 lacks the potential as a cerebral ischemic treatment although it confers certain protection to the cerebral vascular system.

## Introduction

Since hypoxia inducible factor 1 (HIF-1) was discovered as a master regulator in hypoxia about twenty years ago, extensive research has revealed that HIF-1α, the regulatable subunit of HIF-1, is induced in the brain under hypoxic/ischemic conditions [1]. For example, systemic hypoxia, whatever its duration (1, 3, or 6 hours (hrs)), increased the nuclear content of HIF-1α in mouse brain [2]. HIF-1α was significantly induced in rat cerebral cortex after 1 hr of recovery from cardiac arrest and remained elevated for over 12 hrs [3]. A more recent study showed a biphasic activation of HIF-1 after stroke that lasted for up to 10 days [4]. Furthermore, HIF-1α appeared to be mostly induced in the penumbra, the salvageable tissue, in an ischemic brain [5].

Although it is conclusive that ischemia induces the expression of HIF-1, the role of HIF-1 in an ischemic brain is still controversial. On the one hand, HIF-1 regulates the expression of a broad range of genes that facilitate cellular adaptation to low oxygen conditions. Its targets include genes that code for molecules participating in erythropoiesis, cell proliferation, and energy metabolism [6]–[8]. Each of these functions potentially contributes to neuronal survival in ischemia. Indeed, HIF-1 has been reported to protect neurons from apoptosis caused by oxidative stress [9] and focal cerebral ischemia [10]–[12]. Furthermore, neuron-specific knockdown of HIF-1α increased tissue damage and reduced survival rate of mice subjected to middle cerebral artery occlusion (MCAO) [4]. On the other hand, several groups have reported opposite effects of HIF-1 in cerebral ischemia. For instance, Halterman et al. reported that HIF-1α coordinated the activity of p53 in driving ischemia-induced delayed neuronal death instead of providing neuroprotection [13]. Using the same neuron-specific HIF-1α knock-out mice as in the previous study of Baranova *et al.* [4], Helton *et al.* observed that the knock-out of HIF-1α reduced ischemic damage [14].

As a transcription factor, HIF-1 exerts its effects through proteins coded by its downstream genes such as *erythropoietin* (*epo*), *vascular endothelial growth factor* (*vegf*), and *glucose transporter* (*glut*), etc. These downstream genes may express differently and exert different functions in different cell types. For example, VEGF has been reported to have different effects on cell and tissue injuries. On the one hand, it might directly counteract the detrimental neurological effects associated with stroke [15], [16]. VEGF supports the survival of primary motor neurons from hypoxia-induced cell death by binding with neuropilin-1, a receptor known to be involved in axon guidance during development [17]. On the other hand, VEGF promotes blood-brain barrier (BBB) permeability by altering tight junctions under ischemic and inflammatory conditions [18], [19]. Suppressing VEGF by HIF-1 inhibitors improves BBB permeability as observed by Yeh et al [18]. Understanding cell-type dependent effects of HIF-1 will undoubtedly shed new lights on its role in cerebral ischemia and provide potential approaches to promote its beneficial effect and reduce its detrimental function.

**Figure 1:**
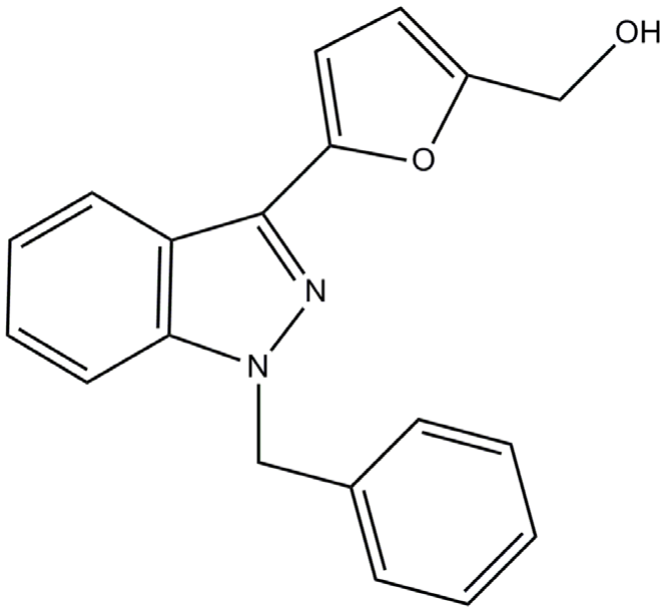
Chemical structure of the HIF-1 inhibitor YC-1.

In the present study, we determined the effects of inhibiting HIF-1 by YC-1 (3-(5'-hydroxymethyl-2'-furyl)-1-benzylindazole, Fig. 1), an established HIF-1 inhibitor, on ischemic outcomes in a rat model of transient cerebral ischemia with the following parameters: infarct volumes and BBB permeability. Furthermore, we studied the effect of YC-1 on the expression of HIF-1 downstream genes *vegf, epo*, and *glut-1, 3* in neurons and brain endothelial cells after cerebral ischemia. The experiments were to reveal the differential effects of HIF-1 in different brain cells in cerebral ischemia. This would provide experimental evidence to understand the intriguing effects of HIF-1 in ischemic stroke.

## Materials and Methods

### Animal model

All procedures using animals were approved by the Institutional Animal Care and Use Committees of University of New Mexico (protocol 05HSC045) and University of Kansas (protocol 192–01) and conformed to the NIH Guidelines for use of animals in research. Male Sprague-Dawley rats, 280–310 g, were from Charles River Laboratory (Wilmington, MA). Animals were maintained in a climate-controlled vivarium with a 12-hr light-dark cycle with free access to food and water. Rats were acclimated to the environment for 7 days before the experiments.

For all surgical and MRI scan procedures, 4.0% isoflurane in N_2_O:O_2_ (70%:30%) was used for anesthesia induction, and 2.0% for anesthesia maintenance. Physiological parameters (e.g., heart rate, respiratory rate, and blood pressure) were monitored during the procedure using a SAII Monitoring System (MRI-Compatible Model 1025, Small Animal Instruments, Inc. Stony Brook, NY). Core (rectal) temperature was maintained at 37.5±0.5°C using a heating pad. Ultra-miniature fiber optic sensors were used to provide minimally invasive, continuous monitoring of blood pressure and heart rate by inserting the optic fiber tip into the left femoral artery.

Middle cerebral artery occlusion (MCAO) followed by reperfusion was conducted using an intraluminal model as previously described [20]. Briefly, external carotid artery (ECA), internal carotid artery (ICA), and pterygopalatine artery of ICA were exposed. A silicone rubber-coated monofilament nylon suture was inserted into the ICA via a slit on the ECA. The suture was advanced along the ICA to the extent of 18 to 19 mm from the bifurcation. Reperfusion was produced by gently withdrawing the suture until the suture tip reached the bifurcation and the incision closed. After surgery, the animals were allowed to recover from anesthesia while being given food and water ad libitum. Buprenorphine was used as post-operative analgesia. For all animals used in this study, successful MCAO was confirmed by laser Doppler flowmetry (LDF) (Moor Instruments, Wilmington, DE) as described in the literature [21]. During ischemia regional cerebral blood flow dropped to 16.2±1.7% of the pre-ischemic level; and after reperfusion the blood flow was restored to 89.5±4.0% of pre-ischemic level. Animals that died during reperfusion were excluded from measuring infarct size, BBB permeability, and HIF-1α expression. Mortality rate was calculated for each group and used as an important parameter for the disadvantageous effect of the HIF-1α inhibitor.

### Experimental groups

Totally 90 Sprague-Dawley (SD) male rats were randomly assigned to the following three groups: YC-1 (without MCAO, n = 5), MCAO (n = 40), and MCAO pretreated with YC-1 (n = 45). In the MCAO (control group) and MCAO + YC-1 groups, 15 rats were used for infarct size measurement by MRI and TTC staining; 15 rats for BBB permeability evaluation; and 10-15 rats for Western blotting and immunostaining (Table 1).

**Table 1:** YC-1-induced mortality of MCAO rats and group sizes for final analyses.

Groups		Initial Group Size	Failed MCAO	Death After Successful MCAO	Group Size for Final Analyses
MCAO	MRI	15	2	1	12
	BBB	15	3	0	12
	HIF	10	0	0	10
	Total	40	5^a^	1 (2.9%)^c^	
YC-1+MCAO	MRI	15	1	5	9
	BBB	15	0	6	9
	HIF	15	0	5	10
	Total	45	1^b^	16 (36.4%)^c^	

a In this MCAO group, four animals were excluded due to a lack of obvious neurological deficits and one due to bleeding during the procedure. ^b^ In YC-1+MCAO group, one was excluded due to a lack of obvious neurological deficits. ^c^ Death rate after successfully completed MCAO.

### Administration of the HIF-1 inhibitor YC-1

**Figure 2:**
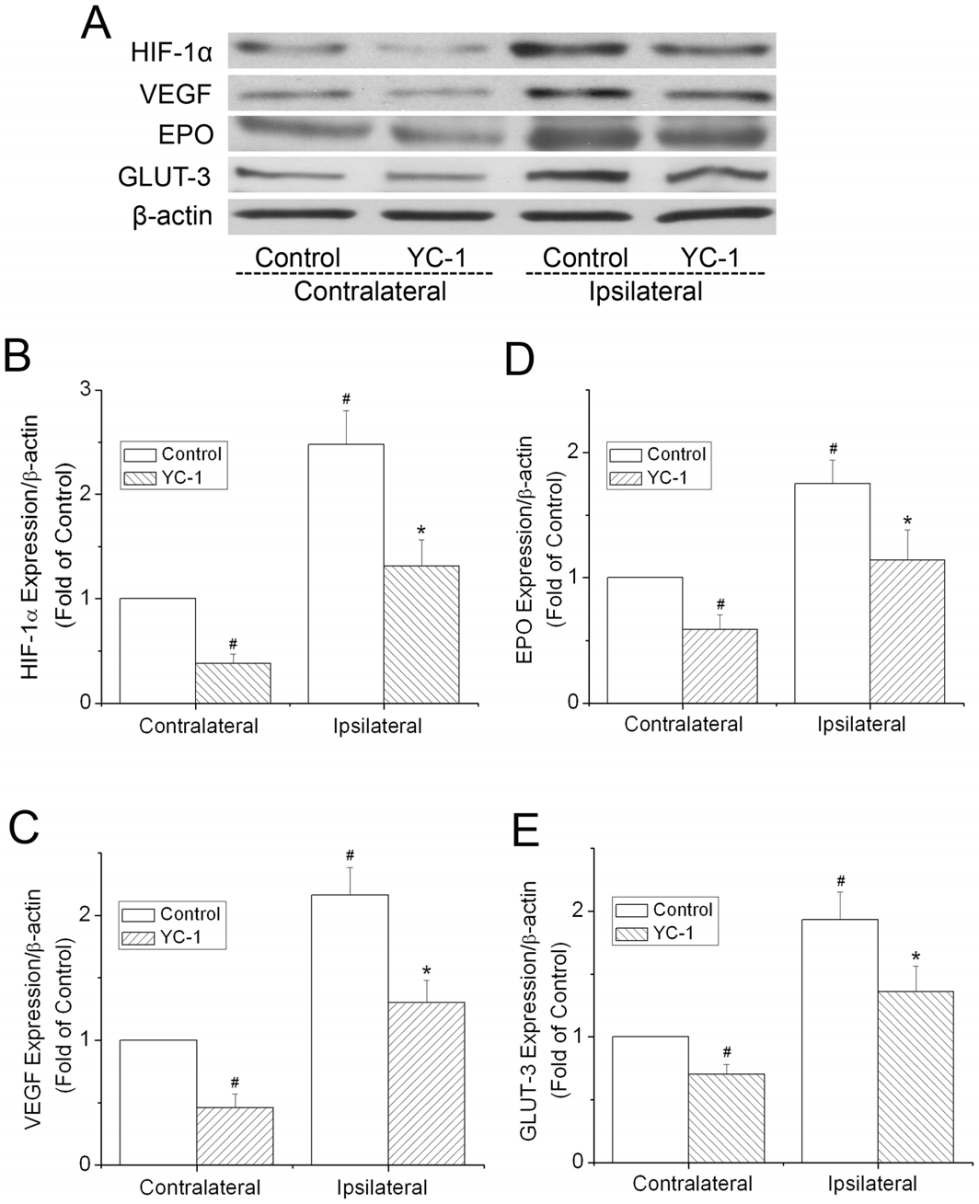
Effect of YC-1 on the expression of HIF-1 in ischemic brains. The protein levels of HIF-1α and its down-stream proteins EPO, VEGF, and GLUT-3 were analyzed by western blotting in brains from rats subjected to 90 min ischemia and 24 hr reperfusion. Rats received YC-1 (2 mg/kg, i.v.) at 24 h and 30 min prior to the onset of ischemia. (**A**) Representative Western blots of HIF-1α and its down-stream proteins. (**B**) Quantification of the HIF-1α protein level. (**C**) Quantification of the VEGF protein level. (**D**) Quantification of the EPO protein level. (**E**) Quantification of the GLUT-3 protein level. Values were normalized to β-actin and contralateral hemispheres of control animals. Values are means ± SEM, n = 5. #*p*<0.05 vs. the contralateral hemisphere of control animals, *p<0.05 vs. the ipsilateral hemisphere of control animals.

YC-1 (Cayman Chemical Company, Ann Arbor, MI), dissolved in a solution of 1% dimethyl sulfoxide (DMSO), was administered at 2 mg/kg body weight through femoral vein at 24 hr and 30 min prior to the onset of ischemia. Rats in control groups received equivolume injections of the DMSO solution. To inhibit HIF-1, researchers have used various dosages of YC-1 ranging from 1 to 30 mg/kg BW [18], [22], [23]. We chose the double injections at 2 mg/kg based on our preliminary analyses of HIF-1α expression after the treatments. As shown in Figure 2, ischemia (MCAO 90 min with 24 h reperfusion) induced a strong up-regulation of HIF-1α in the ipsilateral cortex, and the YC-1 pretreatments markedly inhibited the expression of HIF-1α.

### Western blot analysis

Samples were obtained from the MCA territory cortex on the ischemic sides (ipsilateral) and non-ischemic sides (contralateral) using the corpus callosum as a ventral landmark. Tissues were homogenized in an ice-cold RIPA buffer with 1 µg/ml of a protease inhibitor cocktail (Thermo scientific, Rockford, IL, USA). The homogenates were centrifuged at 14,000 rpm for 15 min at 4°C; and the supernatants were collected. Assays to determine the protein concentration of the supernatants were subsequently performed with a BCA kit (Micro BCA, Pierce). The supernatants were fractionated in an 8% SDS-polyacrylamide gel and transferred to a nitrocellulose membrane. Membranes were blocked in PBS containing 5% nonfat dry milk and 0.01% Tween 20. The membranes were then incubated with different antibodies overnight at 4°C. The primary antibodies for western blotting were HIF-1α (Novus Biologicals, Littleton, CO), VEGF (sc-507, Santa Cruz, Santa Cruz, CA), EPO (sc-7956, Santa Cruz), and GLUT-3 (ab53095, Abcam, Cambridge, MA, USA). Their secondary antibody was goat anti-rabbit IgG-HRP (sc-2030, Santa Cruz). As an internal control, the same membrane was incubated with an antibody specifically for β-actin (Santa Cruz, 1∶1000) after being stripped. Secondary antibody for actin was also the goat anti-rabbit IgG-HRP (sc-2030, Santa Cruz). The blots were detected by chemiluminescence with enhanced chemiluminescence reagent (ECL; Amersham Pharmacia Biotech).

### Measurement of infarct size by magnetic resonance imaging and TTC staining

The rats were transported to the MRI room next to the surgery room at the end of 24-hr reperfusion and placed in the isocenter of the magnet before the imaging session. MRI was performed on a 4.7 T Biospecs MR scanner (Bruker Biospin, Billerica, MA). An actively shielded gradient coil with a 120-cm inner diameter was used. The animals were kept in the same position throughout imaging. For each animal, we performed T2-weighted MRI by using a rapid acquisition with refocused echos sequence. Image data were then transferred to a workstation running Linux for further processing. From the T2-weighted magnetic resonance images, we calculated the volume of infarction using ImageJ.

After MRI scans, the brains were removed and sectioned into 2 mm slices. The slices were incubated in a 2% solution of TTC in 0.1 M PBS (pH 7.4) at 37°C for 30 min and fixed in 10% formalin. TTC staining has been widely used to reflect accurately the extent of irreversible ischemic damage in cerebral tissues in rats [24]. TTC-stained brain sections were photographed using a digital camera (Powershot 400 digital camera, Canon). The infarct size was calculated by the researcher (Saeid Taheri) blind to the treatments given; and the percentage of the infarct area with respect to the total area was digitally quantified by ImageJ. To compensate for the effect of brain edema, the corrected infarct area was calculated as previously described [25]. The edema volume was calculated by measuring the volumes of the affected (V_Ipsi_) and contralateral (V_Contra_) hemispheres and using the formula: edema volume = V_Ipsi_−V_Contra_ [26].

### Immunohistochemical staining

After 24-hr reperfusion, rats were transcardially perfused with ice-cold PBS under anesthesia and then with 4% paraformaldehyde. Brains were isolated and fixed overnight in 4% paraformaldehyde. The brains were then embedded in O.C.T. compound (Sakura Finetek USA, Torrance, CA) and sectioned coronally at 10 µm thickness using a vibrating microtome (Leica Microsystems, Bannockburn, IL). After they were washed and the nonspecific binding sites were blocked with PBS containing 0.05% triton-X100 and 0.25% BSA for 45 min, the brain slices were incubated with primary antibodies against HIF-1α (04–1006, Millipore, Billerica, MA), VEGF (sc-507, Santa Cruz, Santa Cruz, CA), EPO (sc-7956, Santa Cruz), GLUT-3 (ab53095, Abcam, Cambridge, MA, USA), and GLUT-1 (ab652, Abcam, Cambridge, MA) together with the primary antibodies against NEUronal Nuclei (NeuN, MAB377, Millipore) and platelet endothelial cell adhesion molecule-1 (PECAM-1, CBL468, Millipore) in the blocking solution at 4°C overnight. After three washes, the slices were incubated with fluorescent secondary antibodies (donkey anti-rabbit Alexa 488 and goat anti-mouse Alexa 488, Molecular Probes, Carlsbad, CA). After washing, the slices were mounted with Vectashield medium (H-1000, Vector Laboratories, Burlingame). Images were captured under a Leica DMI 4000B fluorescent microscope.

### Determination of blood-brain barrier permeability

Evans blue dye (100 mg/kg, Sigma) was injected into femoral vein 2 hrs after the onset of reperfusion according to a previous report [18]. At the end of hr reperfusion, rats were perfused with saline through the left ventricle until colorless perfusion fluid was obtained from the right atrium. After decapitation, the brain was removed from the skull; and the cortex from each hemisphere was dissected. Samples were weighed and soaked in ml of 50% trichloroacetic acid solution. After homogenization and centrifugation, the extracted Evans blue dye was diluted with ethanol (1∶3); and fluorescence intensity was measured at 620 nm and 680 nm for excitation and emission, respectively, using a fluorescence reader. The tissue content of Evans blue dye was quantified from a linear standard curve derived from known amounts of the dye and was expressed as micrograms per gram of tissues.

### Statistical analysis

The results are presented as means with a standard error of mean. Differences between the groups were established using the least significant difference (LSD) test or ANOVA. Significance was assessed at the p*<*0.05 level.

## Results

### YC-1 suppressed the expression of HIF-1α and its down-stream genes in an ischemic brain

To verify that the YC-1 doses administered to rats were effective in inhibiting HIF-1, protein levels of HIF-1α and its downstream genes, *epo*, *vegf*, and *glut-3*, in the contralateral and the ipsilateral hemispheres of ischemic brains were analyzed by western blotting after the animals were subjected to 90 min MCAO and 24 hr reperfusion. As shown in Figure 2A and 2B, ischemia significantly increased the level of HIF-1α in the ipsilateral hemisphere, compared to the contralateral hemisphere. The increase was remarkably inhibited by YC-1 in both contralateral and ipsilateral hemispheres. Similarly, increases in the expression of VEGF, EPO, and GLUT-3 in ischemic brains were suppressed by the YC-1 treatment (Fig. 2C-E). These results indicate that the doses of YC-1 administration effectively suppressed ischemia-induced expression of HIF-1α and its down-stream genes in the rat model of ischemic stroke.

### YC-1 pretreatment increased death rate of rats after ischemia

YC-1 at 2 mg/kg did not cause death in the five negative control animals (without MCAO). As listed in Table 1, YC-1 significantly increased mortality of MCAO rats. There were 16 deaths in YC-1 treated rats received a successful MCAO, indicating a mortality rate of 36.4% (16/44). Only did one die among the MCAO rats without YC-1 treatments, indicating a mortality rate of 2.9% (1/35).

### YC-1 increased brain tissue damage after ischemia

**Figure 3:**
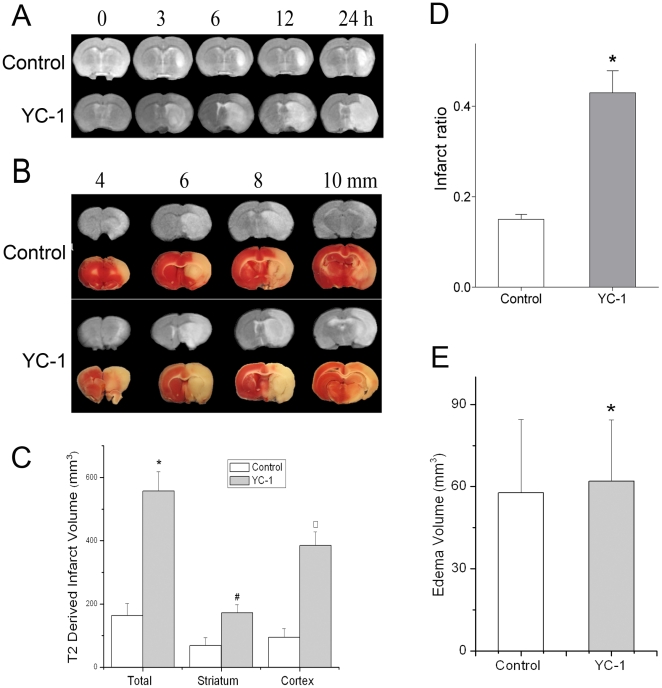
Effect of YC-1 on brain tissue damage of MCAO rats. Brian damage was estimated by MRI and TTC staining after rats were subjected to 90 min ischemia and 24 hr reperfusion. Animals received YC-1 (2 mg/kg, i.v.) at 24 h and 30 min prior to the onset of ischemia. (**A**) Representative MRI images showing time-dependent progression of infarct volumes. T2 weighted MRI images were collected at 0, 3, 6, 12, and 24 hr after MCAO with white area representing infarct area. (**B**) Representative TTC staining (lower panel) and T2 (upper panel) images of brain sections of a MCAO rat. The brain was sectioned from the 4 mm position from the frontal pole and continued in 2-mm interval to 10 mm. (**C**) Quantification of infarct volume with T2-weighted MRI images of rat brain (n = 12 (control), 9 (YC-1)). (**D**) Quantification of brain damage estimated by TTC stained sections (n = 12 (control), 9 (YC-1)). (**E**) Quantification of brain edema volume estimated by TTC stained sections (n = 12 (control), 9 (YC-1)).Values are means ± SEM. *p<0.05 vs. control. #p<0.05 vs. control striatum. ^□^P<0.05 vs. control cortex.

T2 weighted MRI was used to determine the progression of brain tissue damage of MCAO rats. A T2 weighted scan images indicate change and damage in brain tissues [27]. The merit of the MRI is that the images of the brain reflect real-time conditions and allows us to monitor infarct progression in the same animal. To reveal the infarct progression in a MCAO rat, we first collected a series of time-dependent T2 images on the same animals (Fig. 3A). At each time point, the infarct area of YC-1-treated animals was significantly larger that of the control animals (i.e., MCAO without YC-1). Figure 3B demonstrates T2 images of a serial of brain sections after reperfusion for 24 hrs. In each brain section, YC-1 increased the infarct area. The infarct volume was calculated based on the area of hyperintensity in these T2-weighted images. Total, striatal, and cortical infarct volumes of YC-1-treated MCAO rats were 557.4±61.0, 172.2±25.2, and 385.2±43.0 mm^3^, respectively (Fig. 3C). Within the control group, these volumes were 163.4±37.5, 68.7±24.9, and 94.6±27.4 mm^3^, respectively.

We also performed TTC staining, a classic approach in stroke research, to evaluate brain damage caused by MCAO and YC-1. A series of TTC staining of brain sections is shown in Figure 3B. The infarct volume measured by TTC staining demonstrated that YC-1 induced a more severe infarction. The infarct volume in YC-1 pretreated group was 492.8±67.3 mm^3^ vs. 131.6±47.4 mm^3^ in control group. As shown in Figure 3D, the percentages of infarct volume from the whole brain volume in YC-1 and control groups were 43.5±5.6% and 15.1±1.4%, respectively. Brain edema was also calculated after MCAO and 24 hours reperfusion (Fig. 3E). There was no significant difference between YC-1 and control groups in edema volume (62.0±22.3 mm^3^ in YC-1 group vs. 56.7±26.8 mm^3^ in control group).

### YC-1 inhibited ischemia-induced increase in BBB permeability

**Figure 4:**
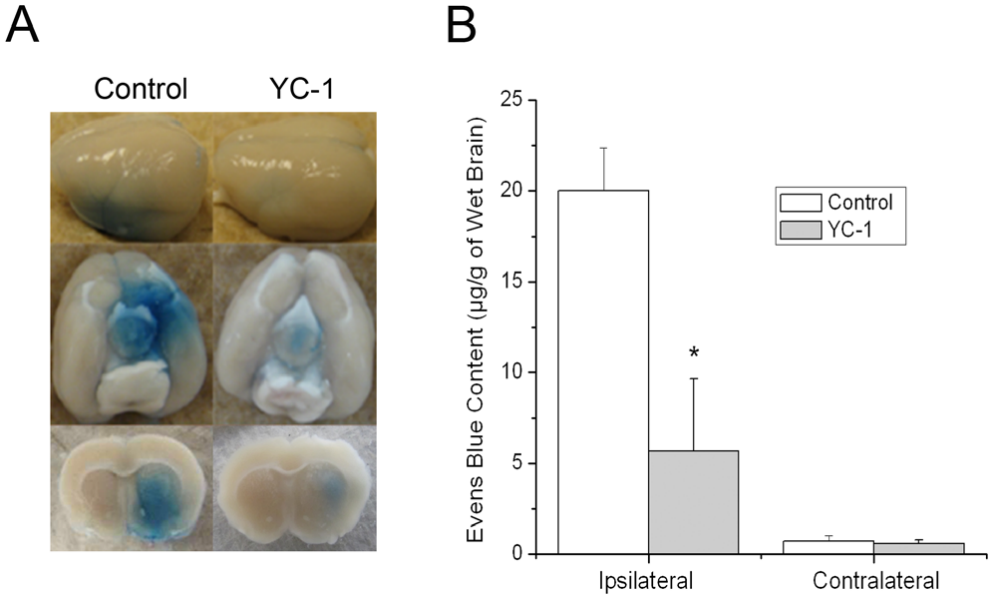
Effects of YC-1 on the BBB permeability of MCAO rats. BBB permeability was estimated by Evans blue leakage after rats were subjected to 90 min ischemia and 24 hr reperfusion. Animals received YC-1 (2 mg/kg, i.v.) at 24 h and 30 min prior to the onset of ischemia. (**A**) Representative images of Evans blue extravasation in a whole brain and coronal sections (bregma +0.70 mm). (**B**) Quantification of Evens blue leakage in ipsilateral and contralateral hemispheres of MCAO rats (n = 12 (control), 9 (YC-1)). Values are means ± SEM. *p<0.05 vs. control.

Evans blue dye was used to serve as a marker of albumin extravasation in evaluating the effect of YC-1 on BBB permeability. Representative images of Evans blue dye in wet brain tissues is shown in Figure 4A. Evans blue leakage increased significantly in the ipsilateral hemisphere (20.0±2.0 µg/g) of brains from rats subjected to 90 min MCAO and 24 hr reperfusion, compared to the contralateral side (0.7±0.3 µg/g). Administration of YC-1 dramatically reduced the Evans blue leakage in the ipsilateral side to 6.0±4.0 µg/g, indicating a 70% reduction (Fig. 4B). These data suggest that YC-1 protected BBB from hyperpermeability induced by MCAO and reperfusion.

### YC-1 inhibited the expression of HIF-1α in neurons in ischemic brains

**Figure 5:**
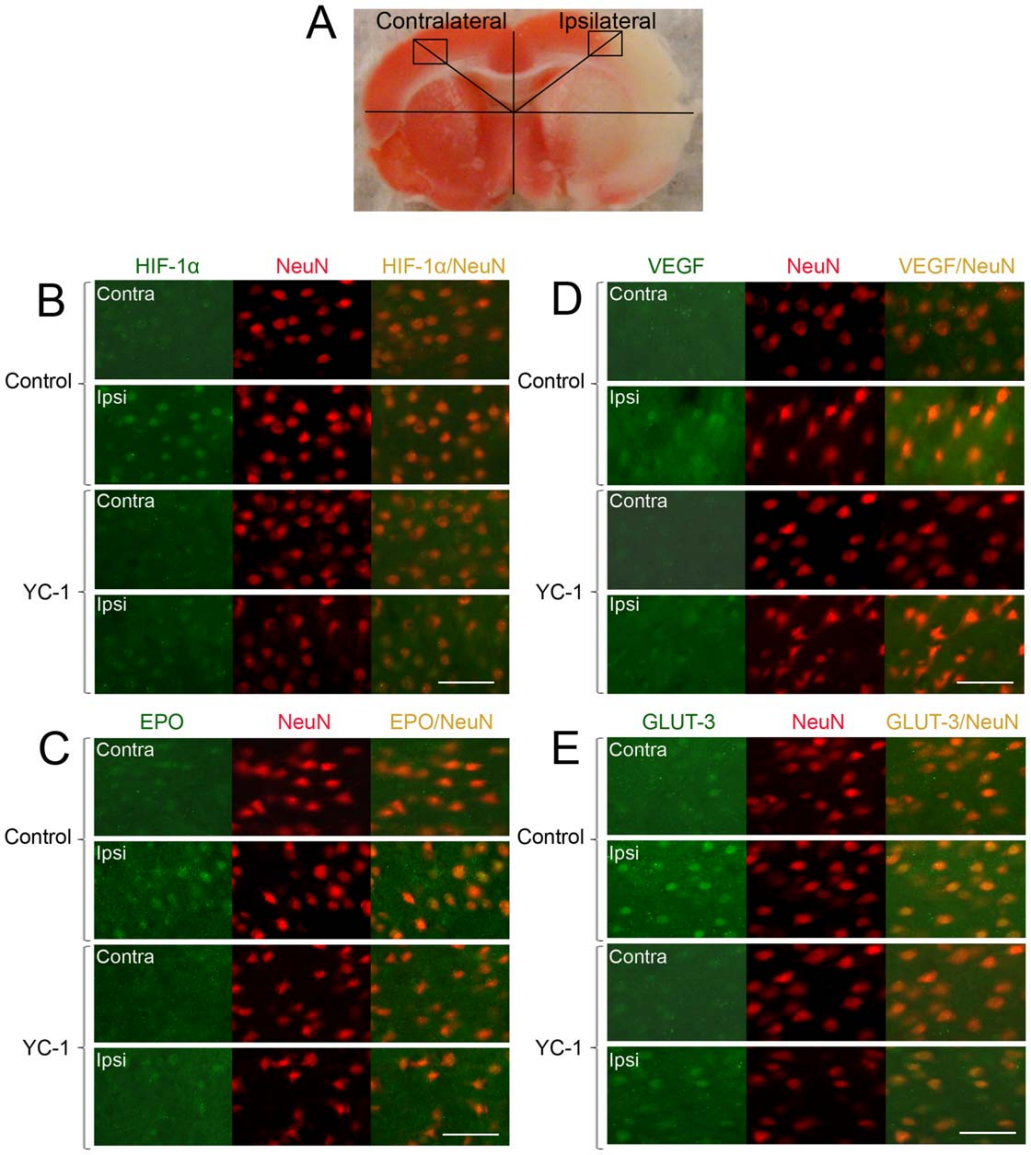
Effect of YC-1 on HIF-1 expression in neurons in ischemic brains. The protein levels of HIF-1α and its down-stream genes were analyzed by double immunostaining with the neuronal marker NeuN after rats were subjected to 90 min ischemia and 24 hr reperfusion. Rats received YC-1 (2 mg/kg, i.v.) at 24 h and 30 min prior to the onset of ischemia. (**A**) TTC-stained rat brain coronal section. Labeled square areas represent locations of immuno images. (**B**) Double immunostaining of HIF-1α (green) and NeuN (red). (**C**) Double immunostaining of EPO (green) and NeuN (red). (**D**) Double immunostaining of VEGF (green) and NeuN (red). (**E**) Double immunostaining of GLUT-3 (green) and NeuN (red). Scale bar, 53 µm.

The above results clearly indicate that inhibiting HIF-1 by YC-1 caused damaging effect on the brain tissue (enlarged infarct) and protective effect on the BBB (improved permeability) determined by Evans blue leakage. To understand the mechanisms responsible for the distinguished effects of YC-1, we analyzed the expression of HIF-1α and its down-stream genes *epo*, *vegf*, and *glut-3* in neurons and endothelial cells in ischemic brains. To explore the neuron-specific expression of HIF-1α and other genes after MCAO with or without the presence of YC-1, double immunohistostaining was performed with neuron marker NeuN. Figure 5A is a typical TTC–stained brain slice and shows the selected locations for imaging. As shown in Figure 5B, the HIF-1α level was extensively up-regulated in neurons in the ipsilateral hemisphere 24 hrs after MCAO. YC-1 significantly reduced HIF-1α expression in the ischemic neurons. The expression of VEGF, EPO and GLUT-3, which are transcriptionally activated by HIF-1, were also significantly increased in the neurons in the ipsilateral hemisphere, compared to that in the contralateral hemisphere (Fig. 5C-E). The staining of the three proteins in the neurons of ipsilateral hemisphere was remarkably reduced by YC-1 treatment. Results from these immunostaining indicate that YC-1 inhibited the expression of HIF-1α and its targeted genes in neurons, which may contribute to the enlarged brain infarct in YC-1-treated MCAO rats.

### YC-1 inhibited the expression of HIF-1α and VEGF in brain endothelial cells

**Figure 6:**
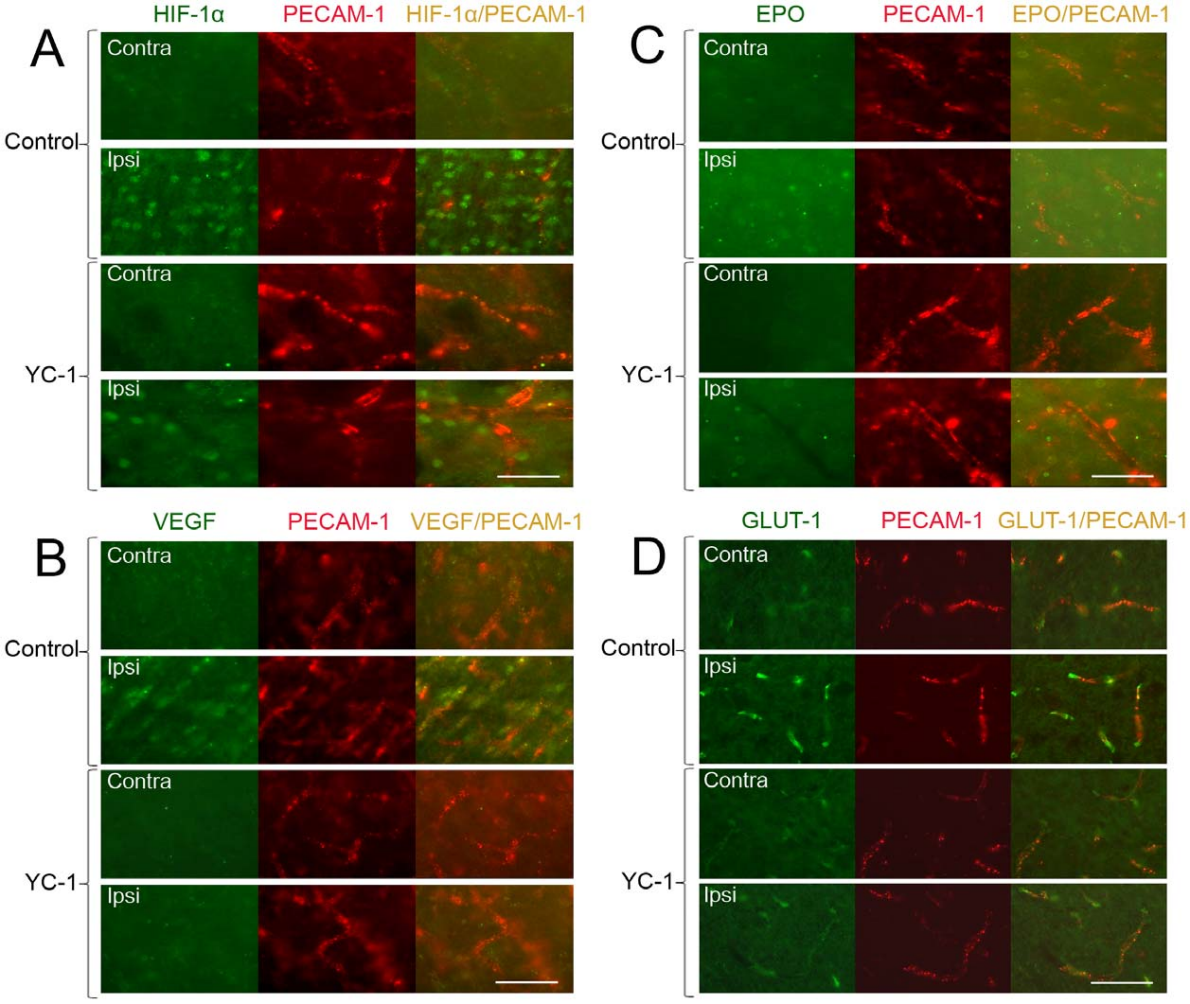
Effect of YC-1 on HIF-1 expression in endothelial cells in ischemic brains. The protein levels of HIF-1α and its down-stream genes were analyzed by double immunostaining with the endothelial marker PECAM-1 after rats were subjected to 90 min ischemia and 24 hr reperfusion. Rats received YC-1 (2 mg/kg, i.v.) at 24 h and 30 min prior to the onset of ischemia. The brain area of interests was the same as in Figure 4. (**A**) Double immunostaining of HIF-1α (green) and PECAM-1 (red). (**B**) Double immunostaining of VEGF (green) and PECAM-1 (red). (**C**) Double immunostaining of EPO (green) and PECAM-1 (red). (**D**) Double immunostaining of GLUT-1 (green) and PECAM-1 (red). Scale bar, 50 µm.

To explore the expression of HIF-1α and its downstream genes after MCAO in brain endothelial cells, double immunohistostaining was performed with the endothelial marker PECAM-1. As shown in Figure 6A, HIF-1α was up-regulated in cells in the ipsilateral hemisphere after 90 min MCAO and 24 hr reperfusion. Some of the HIF-1α positive cells were co-localized with PECAM-1, indicating HIF-1α expression was elevated in endothelial cells. YC-1 significantly decreased the endothelial HIF-1α expression. Figure 6B demonstrates that the VEGF expression was dramatically increased and highly co-localized with endothelial cells in the ipsilateral hemisphere. YC-1 decreased the overall staining of VEGF in both ipsilateral and contralateral hemisphere. The expression of EPO did not seem increased in the endothelial cells of an ischemic brain (Fig. 6C). In addition, the expression of glucose transporter 1 was remarkably increased by ischemia and co-localized with endothelial cells. YC-1 suppressed the glucose transporter expression efficiently. These results indicate that ischemia increased endothelial expression of HIF-1α, which seemed to increase the expression of VEGF and GLUT-1 but not EPO in endothelial cells.

## Discussion

Here we show that ischemia induces the expression of HIF-1 and the proteins coded by its downstream genes *epo*, *vegf*, and *glut* in both neurons and brain endothelial cells. YC-1 is able to suppress the expression. Most significantly, we demonstrate that HIF-1 inhibition by YC-1 has differential effects on brain injury in ischemic stroke (i.e., enlarged infarct volume and improved BBB permeability).

VEGF is the best defined protein that is downstream of HIF-1 in vascular biology [28]. Besides being the most prominent member of the angiogenic growth factor family, VEGF has been known since the 1980s as a vascular permeability factor that increases vascular permeability [29]. More recent studies have shown that VEGF causes brain vascular leakage in pathological conditions such as hypoxia and ischemia [18], [30], [31], possibly by regulating tight junction proteins such as zona occludens 1, claudin-5 [32], [33], and occludin [34]. In this study, we observed significant increase in VEGF expression in neurons and microvessels in an ischemic brain. As shown in Figure 6B, the alignment of VEGF expression highly matches that of the endothelial cells. Inhibiting HIF-1 by YC-1 significantly reduces VEGF expression in brain microvessels that is upregulated by ischemia and subsequently improves BBB permeability. This observation is in line with the previous report by Yeh et al. that suppressing HIF-1 prevents BBB damages [18]. These results are robust in supporting the concept that HIF-1 promotes BBB damage during cerebral ischemia, possible through upregulating VEGF expression. In addition, we observe that the expression of EPO does not co-localize to the expression of PECAM-1. The lack of EPO expression may also contribute to the deterioration of the endothelial cells.

However, this BBB protection resulting from HIF-1 inhibition by YC-1 contributes little to the overall brain tissues injury induced by cerebral ischemia. Our results clearly demonstrate that YC-1 significantly increases brain infarct volume and mortality in the ischemic stroke model. This suggests that the presence of HIF-1 is critical in promoting neuronal survival during ischemia and reperfusion. Among the genes regulated by HIF-1, *epo* and *glut* have extensively studies; and their neuroprotective role has been consistent in the literature [9], [11], [35]–[38]. In agreement with these previous reports, we observe significant upregulation of EPO and GLUT-3 in neurons after ischemia, which is suppressed by YC-1. This result suggests that decreasing EPO and GLUT-3 account for YC-1-mediated exaggeration of brain damage caused by ischemia. Furthermore, VEGF is also upregulated in neurons of an ischemic brain and might counteract detrimental ischemic injuries [15], [16], indicating a complex role of VEGF in different types of cells.

Edema volume has a deleterious impact on the morbidity and mortality after stroke through increasing intracranial pressure and impairing cerebral perfusion and oxygenation during reperfusion [39]. Another piece of evidence indicating that YC-1 cannot ameliorate brain injury after stroke is that YC-1 does not significantly change the edema volume after MCAO and reperfusion (Fig. 3E). This seems contradictory to the results that YC-1 decreases the BBB permeability defined by the extravasation of Evens blue (albumin). The following may explain the seemingly conflicting results. The edema volume (brain swelling) after stroke is mainly determined by the extravasation of water and solutes from plasma due to the increased BBB permeability, which is termed as vasogenic edema [40]. However, BBB has different permeability to water and solutes with different molecular weights [41]. The permeability to larger molecules (e.g. albumin) is easier to be maintained than those with smaller molecular (e.g. sucrose and water) during BBB damage [41]. The ischemia-caused increase in permeability to larger molecules might result from different molecular changes, compared to that to small molecules such as water. It has been reported that another HIF-1 inhibitor, 2ME2, could successfully inhibit the BBB permeability to a soluble protein IgG after MCAO and reperfusion in mice, but only induced a 1% change in the water content of the brain [42]. Our results indicate that inhibiting HIF-1 by YC-1 reduces the permeability to albumin, but it does not change the permeability to water and cannot inhibit the formation of brain edema after stroke.

Giving its potential effects in ischemia, HIF-1 has been a target for understanding mechanisms of cell death and developing novel treatments in stroke. It is of great interests in testing the effect of HIF-1 inhibition and upregulation on brain injuries caused by ischemia. One example of HIF-1 inhibitors is YC-1. Growing evidence suggests that YC-1 exerts an inhibitory effect on the accumulation of HIF-1α induced by hypoxia, iron chelation, and proteasomal inhibition [43]–[45]. YC-1 may directly degrade HIF-1α protein by inducing the degradation of C-terminal of HIF-1α protein [46]. It can also suppress the translation of HIF-1α through PI3K/Akt/mTOR/4E-BP pathway [47]. YC-1 has been reported to inhibit the expression of HIF-1 downstream genes such as *epo* and *vegf* [43]. YC-1 has widely been used as a HIF-1 blocker in research. It has been demonstrated that YC-1 effectively inhibits HIF-1 expression in heart [48], kidney [49], and brain [18]. YC-1 has been shown to reduce disturbances of BBB permeability caused by ischemia by inhibiting HIF-1 expression and suggested as a potential stroke treatment agent [18]. However, the effect of YC-1 on the outcome of cerebral ischemia such as infarct volume has not been tested before. Our data demonstrates that although it ameliorates BBB permeability disturbances caused by ischemia, YC-1 exaggerates ischemic brain damages in terms of infarct volume and mortality. This observation provides novel evidence for the pharmacological effects of YC-1 in ischemic stroke and indicates that YC-1 lacks the potential as a cerebral ischemic treatment although it confers protection to the cerebral vascular system.

It needs to be pointed out that although it is well accepted that YC-1 is an effective HIF-1 inhibitor, it is not a specific HIF-1 suppressor. Besides its effect on HIF-1, YC-1 regulates the intracellular concentration of cGMP though enhancing the activity of soluble guanylate cyclase [50]. Nonetheless, inhibition of soluble guanylate cyclase did not change the effect of YC-1 on blood brain permeability [18]. Furthermore, it has been reported that no serious toxicity was observed in nude mice treated with YC-1 over a 2-week period and that YC-1 has no toxic effect on the normal growth of rat optic nerve and PC12 cells in vitro [44], [51].

A significant focus of stroke research has been on the development of therapeutic strategies that prevent neuronal death and improve recovery. However, to date, few successful therapeutic strategies have emerged. HIF-1, as a gene transcriptional regulator induced in hypoxia, has been discussed at length for its roles in brain tissues during ischemia. Although detrimental effects of HIF-1 have been observed in ischemic brains, regulating HIF-1α induction and the genes induced by HIF-1 is a highly promising therapeutic strategy for cerebral ischemia [6], [52]-[54] due to their endogenous adaptive responses to hypoxia and ischemia. HIF-1 induces expressions of a wide range of genes; and the induction and functions of these genes may depend on the specific cell types. As demonstrated in this study, HIF-1 may function differently in different cells. Future studies need to focus on specific types of cells and cellular targets to better understand the role of HIF-1 in stroke as well as other pathological conditions.

In summary, our results provide novel evidence that HIF-1 function differently in different cells depending on the functions of the proteins coded by its downstream genes in the specific type of cells. The results also indicate that YC-1 lacks the potential as a cerebral ischemic treatment although it confers protection to the cerebral vascular system.
